# A New Strategy to Investigate RNA:DNA Triplex Using Atomic Force Microscopy

**DOI:** 10.3390/ijms25053035

**Published:** 2024-03-06

**Authors:** Giovanni Merici, Davide Amidani, Giorgio Dieci, Claudio Rivetti

**Affiliations:** Department of Chemistry, Life Sciences and Environmental Sustainability, University of Parma, 43124 Parma, Italy; giovanni.merici@unipr.it (G.M.); d.amidani@chiesi.com (D.A.); giorgio.dieci@unipr.it (G.D.)

**Keywords:** lncRNA, RNA:DNA triplex, atomic force microscopy

## Abstract

Over the past decade, long non-coding RNAs (lncRNAs) have been recognized as key players in gene regulation, influencing genome organization and expression. The locus-specific binding of these non-coding RNAs (ncRNAs) to DNA involves either a non-covalent interaction with DNA-bound proteins or a direct sequence-specific interaction through the formation of RNA:DNA triplexes. In an effort to develop a novel strategy for characterizing a triple-helix formation, we employed atomic force microscopy (AFM) to visualize and study a regulatory RNA:DNA triplex formed between the Khps1 lncRNA and the enhancer of the proto-oncogene *SPHK1*. The analysis demonstrates the successful formation of RNA:DNA triplexes under various conditions of pH and temperature, indicating the effectiveness of the AFM strategy. Despite challenges in discriminating between the triple-helix and R-loop configurations, this approach opens new perspectives for investigating the role of lncRNAs in gene regulation at the single-molecule level.

## 1. Introduction

Chromatin-associated non-coding RNAs (ncRNAs) represent an extremely numerous yet still underexplored class of macromolecules with the potential to influence gene expression at various levels [1]. Many of them were shown in recent years to mediate the recruitment of chromatin modifiers to specific genomic loci, thereby profoundly influencing genome organization and regulation [2,3]. A key aspect of the involvement of chromatin-associated ncRNAs in gene regulation is the mechanism of their locus-specific binding to DNA. The two main modes of RNA tethering to chromatin are based either on the non-covalent interaction with chromatin-associated proteins [4] or the direct, sequence-specific interaction of the RNA with DNA through base pairing [5,6]. In turn, the sequence-specific pairing of RNA to DNA may occur via the formation of either R-loops, triple-stranded structures containing a DNA:RNA hybrid and a displaced DNA strand [7], or RNA:DNA triple helices, with the RNA bound to the major groove of the DNA double helix using the Hoogsteen base pairing [5,8]. Both R-loops and RNA:DNA triplexes have recently been shown to be endowed with great gene regulatory potential [5,7] but also with the potential to induce genome instability [9,10]. An important difference between the two types of RNA-dependent DNA targeting is that while R-loop formation requires local DNA melting and strand displacement, the RNA:DNA triplex formation directly relies on the interaction of a triple-helix forming sequence provided by an RNA molecule with a target site in the major groove of double-stranded DNA, in a manner reminiscent of sequence-specific DNA recognition using transcription factors. Triple-helix target sites typically consist of a tract of DNA duplex with a skewed distribution of purines and pyrimidines in the two strands so that the purine-rich strand is available for Hoogsteen base pairing with the third strand of RNA. While a pyrimidine-rich RNA sequence binds to the major groove of the DNA target in parallel orientation to the polypurine DNA strand, a purine-rich RNA sequence will pair in the opposite direction [5]. Importantly, suppose the target DNA site lies in a gene regulatory region, e.g., a promoter or an enhancer element. In that case, a triplex can modulate its activity either by directly inducing a local conformational/topological change [11,12] or, more frequently, by recruiting regulatory proteins via interaction with the ncRNA portion protruding from the triplex [13,14,15,16,17]. Although the ability of triplex-forming ncRNAs to recruit epigenetic regulators site-specifically has been documented in several in vivo studies, attempts to recapitulate these regulatory interactions in vitro have lagged behind. In particular, while the RNA:DNA triplex formation has been largely characterized by testing triplex-forming RNA oligonucleotides in bulk biochemical assays like the electrophoretic mobility shift assay (EMSA), single molecule methods, allowing to directly observe higher-order macromolecular assemblies and to appreciate their structural heterogeneity, have never been applied to regulatory RNA:DNA triplexes. As a first step towards the single-molecule characterization of chromatin complexes involving triplex-forming ncRNAs, we devised a strategy to visualize and study via atomic force microscopy a previously characterized regulatory triple helix formed between a homopyrimidine sequence stretch in the Khps1 lncRNA and a homopurine target site in the enhancer of the proto-oncogene *SPHK1* [17,18]. The rationale for choosing this system was to test our newly devised approach on a regulatory interaction with a previously documented involvement in key cellular processes that bulk approaches had thoroughly characterized.

## 2. Results

AFM is a powerful imaging technique that provides high-resolution images of nucleic acids (DNA and RNA) and protein–nucleic acid complexes deposited onto a flat surface, generally mica [19,20]. While AFM has been employed to image and study triplex DNA molecules [21,22,23], the visualization of RNA:DNA triple helices poses a technical challenge. The poor lateral resolution of AFM hinders the effective visualization of small RNA molecules (20–30 nucleotides long), such as those previously used for the in vitro characterization of regulatory triple helices [11,17]. As an experimental strategy to overcome this limitation, we planned to use, as a triplex-forming RNA, an RNA probe containing a double-stranded extension conferring a rod-like configuration with dimensions sufficient for easy visualization in AFM images. To this end, we designed a 248 bp double-stranded RNA harboring a 22 nt 3′ overhang with the pyrimidine stretch reported to form a triple helix with the TFR2 enhancer sequence of SPHK1 [17].

### 2.1. Synthesis of the Target DNA and the RNA Probes

**Figure 1:**
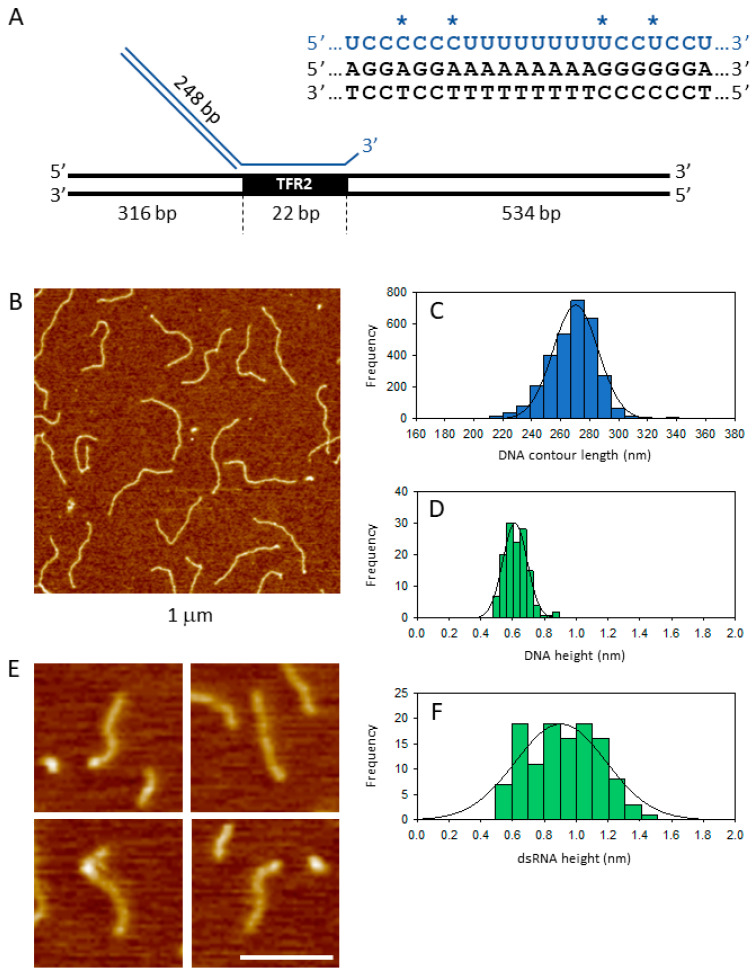
(**A**) Schematic representation of the RNA:DNA triplex construct analyzed in this study. RNA is drawn in blue, while DNA is in black. Stars point to RNA:DNA mismatches. (**B**) AFM image of the 872 bp DNA template containing the TFR2 target sequence and (**C**) DNA contour length distribution of 3069 free DNA molecules fitted with a Gaussian function with mean 269 ± 18 nm. (**D**) Distribution of image profiles for 132 DNA molecules fitted with a Gaussian function with a mean of 0.61 ± 0.08 nm. (**E**) Gallery of dsRNA fragments used to form the triple helix. (**F**) Distribution of image profiles of 119 dsRNA molecules fitted to a Gaussian distribution with mean 0.90 ± 0.29 nm. Scale bar 100 nm.

A dsDNA fragment encompassing the upstream region of the *SPHK1* gene and containing the Khps1 lncRNA target site in its natural sequence context was prepared via PCR amplification from human genomic DNA. The 872 bp DNA amplicon featured the TFR2 sequence at 316 bp from the upstream and 534 bp from the downstream (Figure 1A). In the case of triple-helix formation, this asymmetrical arrangement allows identification from the AFM images of the upstream and downstream DNA arms and the dsRNA probe. Figure 1B,C display a representative AFM image of the DNA target templates and the contour length distribution of 3069 free target DNA molecules, fitted to a Gaussian function with a mean 269 nm and SD ± 18 nm. This value agrees with previous AFM measurements of B-form DNA using AFM [24]. Figure 1D depicts the image profile distribution of DNA molecules, revealing a DNA mean height of 0.61 ± 0.08 nm. It is worth noting that this measurement appears lower than the canonical value of 2 nm for B-form DNA, likely due to the compression effect induced by the tapping tip during imaging.

For in vitro synthesis of the dsRNA probe with the 22 nucleotides pyrimidine stretch contained in the lncRNA Khps1, we constructed two DNA templates capable of producing, via T7 RNA polymerase-dependent transcription, complementary RNAs except for the pyrimidine sequence at the 3′ end.

Specifically, an RNA filament of 273 nucleotides, containing the 3′-terminal triple-helix forming sequence (ssRNA1) and a 248 nucleotides complementary RNA filament (ssRNA2), were separately synthesized, purified, and annealed to obtain a double-stranded RNA probe in which the 3′-terminal triple-helix forming sequence is left unpaired. Figure 1E shows a gallery of the dsRNA molecules obtained from the annealing reaction. They appear as rod-like filaments with a length appropriate for a 250 bp dsRNA and a white globular feature at one end, likely representing the collapsed single-stranded tail. As depicted in Figure 1F, A-form dsRNA appears thicker than dsDNA (mean height 0.90 ± 0.29 nm), a feature that can be used to distinguish the RNA probe from the DNA template. This type of RNA probe, as well as the ssRNA1 and ssRNA2 alone, were used in the subsequent triple-helix formation and analysis experiments.

### 2.2. Triple-Helix Formation and AFM Imaging

For the triple helix formation, the 872 bp DNA fragment comprising the TRF2 site was incubated with either dsRNA or ssRNA probes under different pH and temperature conditions, as detailed in Materials and Methods. After an incubation time of about 1 h, the reaction was deposited on freshly cleaved mica, rinsed with Milli-Q water, and dried with a stream of nitrogen.

**Figure 2:**
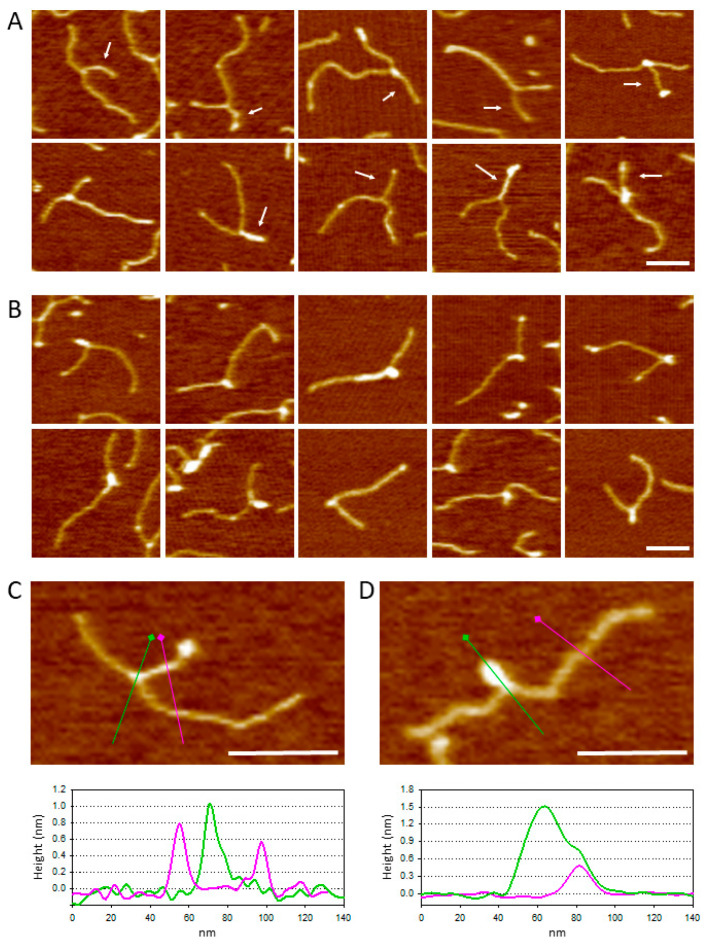
(**A**) Gallery of RNA:DNA triplex formed with dsRNA fragments harboring the 22 pyrimidine TFR2 stretch at the 3′ end. White arrows point to RNA as determined by the contour length. (**B**) Gallery of RNA:DNA triplex formed with ssRNA harboring the 22 pyrimidine TFR2 stretch at the 3′ end. (**C**) Image of a triple-helix complex as in A together with the image profiles obtained along the colored lines. (**D**) Image of a triple-helix complex as in (**B**) together with the image profiles obtained along the colored lines. Filled square symbols represent the starting point of the profiles. Bars size 100 nm.

Figure 2 shows a gallery of triple-helix complexes visualized using AFM in which the RNA moiety is bound to DNA at a position compatible with that of the TFR2 target site. The complexes shown in Figure 2A likely result from the formation of a triple helix between the TFR2 target sequence and the single-stranded pyrimidine stretch of the dsRNA probe. The dsRNA probe can be distinguished by its length, slightly smaller than the shorter DNA arm. The complexes shown in Figure 2B likely result from the formation of a triple helix between the TFR2 target sequence and the pyrimidine stretch of the ssRNA1 probe. Because of its higher flexibility, the ssRNA probe is typically visualized as a collapsed globular feature rather than a rod-like filament. In both instances, specific RNA:DNA triplexes were identified based on their position along the DNA template. Namely, only complexes with a DNA contour length within ±1 SD from the mean (269 ± 18 nm) and with an arm ratio in the range of 0.6 ± 0.1 were considered specific. We noticed that, in several cases, the formation of the triplex induces a bend in the DNA template. However, conducting a quantitative AFM analysis of DNA bending presents challenges because of potential distortions in the deposition process. Consequently, this study did not pursue an analysis of bend angles.

**Figure 3:**
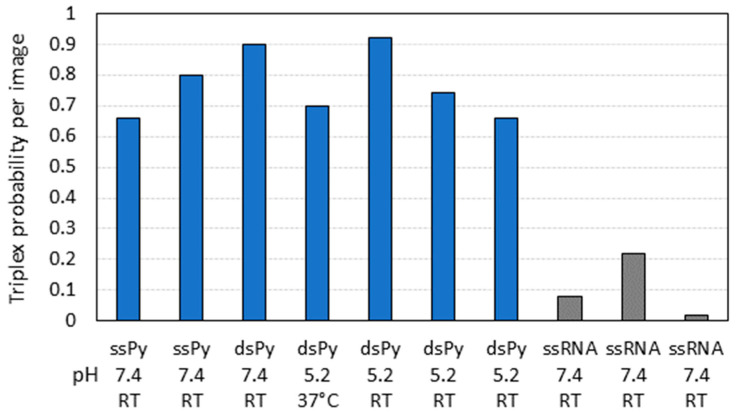
Bar plot representation of the triplex probability per image (number of RNA:DNA triplex scored divided by the total number of images) in different experiments conducted under different conditions of pH and temperature (blue bars). ssPy: single-stranded RNA with TFR2 pyrimidine stretch at the 3′ end; dsPy: double-stranded RNA with TFR2 pyrimidine stretch at the 3′ end; ssRNA: single-stranded RNA without the TFR2 pyrimidine stretch used as control (gray bars).

To quantitatively assess the formation of the triple helix, we counted the number of DNA molecules exhibiting either a rod or a globular structure, specifically bound at the TFR2 site of the DNA template. Experiments were conducted under different conditions of temperature and pH using either dsRNA or ssRNA1 probes, both with the pyrimidine stretch at the 3′ end. ssRNA2, lacking the TFR2 pyrimidine stretch, was used as a negative control. For each experiment, a set of 50 images, with a scan size of 2 μm, were collected and analyzed. The bar plot in Figure 3 reports the results of all the experiments performed. A total of 794 complexes were scored, of which only 286 were deemed specific: 226 complexes had a visible linear RNA interacting with the target DNA, while 60 complexes displayed a globularly shaped RNA at the TFR2 site. 

The data suggest that the strategy devised for visualizing the formation of the RNA:DNA triple helix using AFM is effective. Indeed, the observation reveals a significantly higher number of complexes formed compared to the control in all the experiments conducted. Interestingly, there is no observable dependence of the number of complexes on the conditions of triple-helix formation, which occurs consistently at pH 5.2 and 7.4. Additionally, the reaction does not appear to be influenced by variations in temperature, although only one experiment was conducted at 37 °C and pH 5.

## 3. Discussion

In this study, a complex between a fragment of a regulatory lncRNA and its target site on DNA is visualized at the single-molecule level for the first time. Although the study has shown that triple-helix formation is possible using both ssRNA and dsRNA probes, the use of a dsRNA construct such as the one proposed in this study has several advantages. First, recognizing specific complexes is straightforward since the Y shapes formed are easily discernible from other structures. Second, the fact that the single-stranded RNA sequence is only the terminal stretch of dsRNA prevents the formation of nonspecific contacts between RNA and DNA. Furthermore, the use of a dsRNA probe was expected to facilitate the discrimination between the triple helix and R-loop. As stated above, the triple helix formation with the polypyrimidine RNA sequence occurs through a Hoogsteen pairing with the polypurinic strand of the DNA target, where the strands are arranged in a parallel orientation. Conversely, the formation of an R-loop still involves the purine strand of the DNA target but through a Watson–Crick pairing, where the strands are arranged in an antiparallel manner. The off-centered position of the TFR2 sequence was anticipated to enable the distinction between these two types of interactions, as it would lead to the formation of two distinct Y-shaped configurations as those exemplified in the following scheme (see also Figure 1A). 

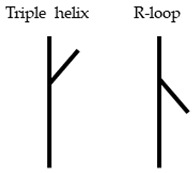



Unfortunately, the data we collected did not allow for such discrimination because we found that the RNA probe did not exhibit a preferred orientation. In other words, the triple-helix junction of the RNA probe was too flexible and did not reflect the orientation of the pyrimidine stretch in the major groove. Another difficulty lies in the fact that, due to the lower binding strength of Hoogsteen pairings compared to Watson–Crick pairings [25], the frequency at which triple helices are visualized is low (one or two per image). This limitation prevents obtaining a statistically significant number of complexes for conducting this type of analysis.

Although the RNA probes used in this study contain only a sequence of 22 nucleotides from the larger Khps1 lncRNA, the analysis has the potential to extend this approach to RNAs encompassing most or the entire sequence of a lncRNA. This is especially relevant for the possibility of studying the interaction between lncRNA and nuclear regulatory proteins at a single-molecule level. In particular, the lncRNA-mediated recruitment of chromatin modifiers to target gene promoters might represent a key mechanism of eukaryotic gene regulation, yet it still deserves investigation through rigorous methodologies [3]. How a lncRNA tethered to a specific DNA target site can promote protein recruitment is still largely unexplored. With this respect, our approach should allow us to investigate whether the triple-helix formation serves merely as a tethering mechanism or if it exerts influence on the recruitment process. For instance, it could be examined whether the protein must engage in simultaneous interactions with both DNA and RNA to facilitate specific functions within the complex. In general, even though the function of lncRNA in chromatin regulation is likely to involve complex interactions within higher-order nuclear architecture, the single-molecule in vitro studies of lncRNA biochemical properties, made possible using our approach, could fruitfully complement both techniques to detect protein–lncRNA interactions in vivo [26] and bulk methods to study lncRNA–protein interactions in solution [27].

## 4. Materials and Methods

### 4.1. Cloning and Purification of the TFR2 DNA Template

The target DNA fragment containing the 22 bp TFR2 sequence was obtained from human genomic DNA. A DNA fragment of 872 bp containing the upstream sequence of the *SPHK1* gene (genomic location 568815581:76381567-76387860 *Homo sapiens* chromosome 17, GRCh38.p2 Primary Assembly) was amplified via PCR using primers TH01 and TH02 (Table 1), each containing an *EcoRI* restriction site near the 5′ end to facilitate cloning. The amplicon was digested with *EcoRI* and ligated to a linearized pNEB193 plasmid to generate a pSPHK1 plasmid. The 872 bp DNA template used for AFM imaging was obtained via PCR using pSPHK1 as a template and primers TH01 and TH02 under standard reaction conditions. The amplicon was gel-purified using electrodialysis followed by phenol–chloroform extraction and ethanol precipitation. The DNA was resuspended in a low TE buffer (10 mM Tris-HCl pH 8, 1 mM KCl, and 0.1 mM EDTA) and stored at 4 °C.

### 4.2. Synthesis of the TFR2 RNA Probes

The ssRNA and dsRNA probes used for the formation of the triplex were obtained in vitro via T7 RNA polymerase run-off transcription using DNA templates obtained with PCR from the pET-28b plasmid. Each DNA template contains a T7 promoter at one end, from which the transcription of the two complementary RNAs can be initiated. The PCR reaction with primers TH07 and TH03 produced a DNA amplicon of 295 bp, while the PCR reaction with primers TH06 and TH08 produced a DNA amplicon of 270 bp. After PCR, both amplicons were purified with a clean-up kit (Fisher Molecular Biology, Trevose, PA, USA). A T7 transcription of the 295 bp DNA resulted in a 273 nt RNA (ssRNA1), which contains a stretch of 22 pyrimidines at the 3′ (see TH07 primer sequence). The T7 transcription of the 270 bp DNA resulted in a 248 nt RNA (ssRNA2) that was complementary to ssRNA1 except for the terminal pyrimidine stretch.

Transcription was carried out using the Invitrogen MAXIscript T7 kit (Thermo Fisher Scientific, Cleveland, OH, USA) for 1 h at 37 °C. TURBO DNase was used at the end of the reaction to remove any residual DNA template. The DNase reaction was carried out at 37 °C for 15 min and stopped by the addition of 1 μL of 0.5 mM EDTA. The RNA was purified using phenol–chloroform extraction and ethanol precipitation. Both RNAs were resuspended in Milli-Q water treated with diethylpyrocarbonate (DEPC), and RNA concentration was measured with the NanoDrop One spectrophotometer (Thermoscientific, Rochester, NY, USA). The dsRNA probe was obtained by annealing ssRNA1 and ssRNA2 as follows: ssRNA1 and ssRNA2 at an equimolar concentration of 133 nM were mixed in 10 mM KCl, 1 mM MgCl_2_, and the reaction was heated at 90 °C for 5 min in the thermo-block and let it cool until room temperature was reached (~1 h).

### 4.3. Triplex Formation and Atomic Force Microscopy

RNA:DNA triplex complexes were assembled under different conditions depending on the experiment. In all cases, the reaction was performed by mixing a 40 nM DNA target template with a 50 nM RNA probe (dsRNA, ssRNA1, or ssRNA2). For experiments at pH 7.4, the reaction was assembled in 50 mM HEPES pH 7.4, 50 mM MgCl_2_, and 100 mM KCl and incubated at room temperature for 30 min. The reaction was then diluted 1:20 in 20 μL of a deposition buffer (10 mM HEPES pH 7.1, 5 mM MgCl_2_, and 10 mM NaCl) and immediately deposited onto freshly cleaved mica. For experiments at pH 5.2, the reaction was assembled in 20 mM sodium acetate pH 5.2, 50 mM MgCl_2_, and 100 mM KCl. The reactions were incubated at room temperature or at 37 °C for 30 min. The reaction was then diluted 1:20 in 20 μL of a deposition buffer (5 mM sodium acetate pH 5.0 and 10 mM MgCl_2_) and immediately deposited onto freshly cleaved mica. In all experiments, the mica disk with the deposition drop was incubated for 2 min, rinsed with Milli-Q water, and dried with a stream of nitrogen. All buffers were prepared in Milli-Q water treated with DEPC. AFM imaging was carried out “in air” with a Nanoscope IIIA microscope (Digital Instruments, Santa Barbara, CA, USA) operating in tapping mode and equipped with the E scanner. Commercial silicon cantilevers with a nominal tip radius of curvature < 10 nm and a resonance frequency of 110–220 kHz were purchased from MikroMasch or NuNano. For each experiment, a set of 50 images (512 × 512 pixels) with a scan size of 2 μm were collected.

### 4.4. Image Analysis

To identify specific RNA:DNA triplex complexes, we mapped the position of the bound RNA along the contour of the target DNA. The contour length of the DNA target was measured as previously described [24]. Utilizing the contour length distribution of free DNA molecules (Figure 1C), only complexes with a DNA contour length within ±1 SD from the mean (269 ± 18) were included in the analysis. The position along the contour of the bound RNA was manually selected by clicking the center of the interaction with the computer mouse, allowing the determination of the contour length of the two DNA arms. Based on the DNA construct illustrated in Figure 1A, a triple-helix complex was classified as specific if the short-arm/long-arm ratio was in the range of 0.6 ± 0.1. Complexes that did not comply with these conditions were discarded from the analysis.

## Figures and Tables

**Table 1:** Primers used in this study.

Primer	Sequence
FA01 TH01	ATATGAATTCAATGACAAGAATGAGGG
FA02 TH02	ATATGAATTCCTCCGAGAAACAGGAAC
FA07 TH07	GCCCCAGGAGGAAAAAAAAAGGGGGGATTAGCAGCCGGATCTCAGTGG
FA03 TH03	GAAATTAATACGACTCACTATAGGGGAATTGTG
FA08 TH08	GAAATTAATACGACTCACTATAGGGTTAGCAGCCGGATCTCAGTGG
FA06 TH06	GGGAATTGTGAGCGGATAACAATTCC

## Data Availability

All data underlying the results are available as part of the article and no additional source data are required.
